# Biochemical Diagnosis of Catecholamine-Producing Tumors of Childhood: Neuroblastoma, Pheochromocytoma and Paraganglioma

**DOI:** 10.3389/fendo.2022.901760

**Published:** 2022-07-26

**Authors:** Graeme Eisenhofer, Mirko Peitzsch, Nicole Bechmann, Angela Huebner

**Affiliations:** ^1^ Institute of Clinical Chemistry and Laboratory Medicine, Universitätsklinikum Carl Gustav Carus Dresden, Technische Universität Dresden, Dresden, Germany; ^2^ Department of Internal Medicine III, Universitätsklinikum Carl Gustav Carus Dresden, Technische Universität Dresden, Dresden, Germany; ^3^ Department of Pediatrics, Universitätsklinikum Carl Gustav Carus Dresden, Technische Universität Dresden, Dresden, Germany

**Keywords:** pediatric, pheochromocytoma, paraganglioma, neuroblastoma, catecholamines, metanephrines, methoxytyramine, homovanillic acid

## Abstract

Catecholamine-producing tumors of childhood include most notably neuroblastoma, but also pheochromocytoma and paraganglioma (PPGL). Diagnosis of the former depends largely on biopsy-dependent histopathology, but this is contraindicated in PPGL where diagnosis depends crucially on biochemical tests of catecholamine excess. Such tests retain some importance in neuroblastoma though continue to largely rely on measurements of homovanillic acid (HVA) and vanillylmandelic acid (VMA), which are no longer recommended for PPGL. For PPGL, urinary or plasma metanephrines are the recommended most accurate tests. Addition of methoxytyramine to the plasma panel is particularly useful to identify dopamine-producing tumors and combined with normetanephrine also shows superior diagnostic performance over HVA and VMA for neuroblastoma. While use of metanephrines and methoxytyramine for diagnosis of PPGL in adults is established, there are numerous pitfalls for use of these tests in children. The establishment of pediatric reference intervals is particularly difficult and complicated by dynamic changes in metabolites during childhood, especially in infants for both plasma and urinary measurements, and extending to adolescence for urinary measurements. Interpretation of test results is further complicated in children by difficulties in following recommended preanalytical precautions. Due to this, the slow growing nature of PPGL and neglected consideration of the tumors in childhood the true pediatric prevalence of PPGL is likely underappreciated. Earlier identification of disease, as facilitated by surveillance programs, may uncover the true prevalence and improve therapeutic outcomes of childhood PPGL. For neuroblastoma there remain considerable obstacles in moving from entrenched to more accurate tests of catecholamine excess.

## Introduction

Catecholamine-producing tumors include neuroblastoma, as well as pheochromocytoma and paraganglioma (PPGL), all derived from cells of the neural crest. Neuroblastoma occurs almost exclusively in childhood and originate from immature embryonic neuroblast cells that undergo transformation to form tumors at intra-adrenal and extra-adrenal locations. PPGL similarly occur at respective intra-adrenal and extra-adrenal locations, but originate from chromaffin cells or their chromoblast precursors, and are usually detected in adulthood though may be overlooked in childhood.

It is now apparent that neuroblasts, sympathoblasts, chromoblasts and mature chromaffin cells originate from neural crest derived Schwann cell precursors by way of different transitions and manifest by variable stages of differentiation (1, 2). These emerging concepts about development from different neural crest derivatives are fundamental to a complete understanding of the utility of catecholamine-related biomarkers for diagnosis of neuroblastoma and PPGL. In particular, differences in transition of neural-crest derived tumor precursors appear to be recapitulated in the considerable heterogeneity of presentations of both neuroblastoma and PPGL, including the nature of catecholamine production.

While neuroblastomas are characterized by poorly developed catecholamine biosynthetic and secretory pathways, there is nevertheless variation in this that relates to differences in catecholamine-associated features important for biochemical testing and disease aggressiveness (3–6). PPGL on the other hand show more mature catecholamine biosynthetic and secretory pathways compared to neuroblastoma, though even within those two intra- and extra-adrenal groups of tumors there is considerable heterogeneity in differentiation that impacts the nature of catecholamine secretory and metabolic products employed for biochemical diagnosis (7).

Of relevance to this article, PPGL detected in childhood and young adulthood tend to be poorly differentiated and often occur secondary to a particular group of mutated genes (i.e., so called cluster 1 group mutations) that predispose to more often extra-adrenal paraganglioma and multifocal adrenal and extra-adrenal tumors than the tumors that originate from cluster 2 group mutations (7–9). As detailed later, such differences have relevance to biochemical testing strategies and interpretation of biochemical test results in children with suspected chromaffin cell tumors.

**Figure 1:**
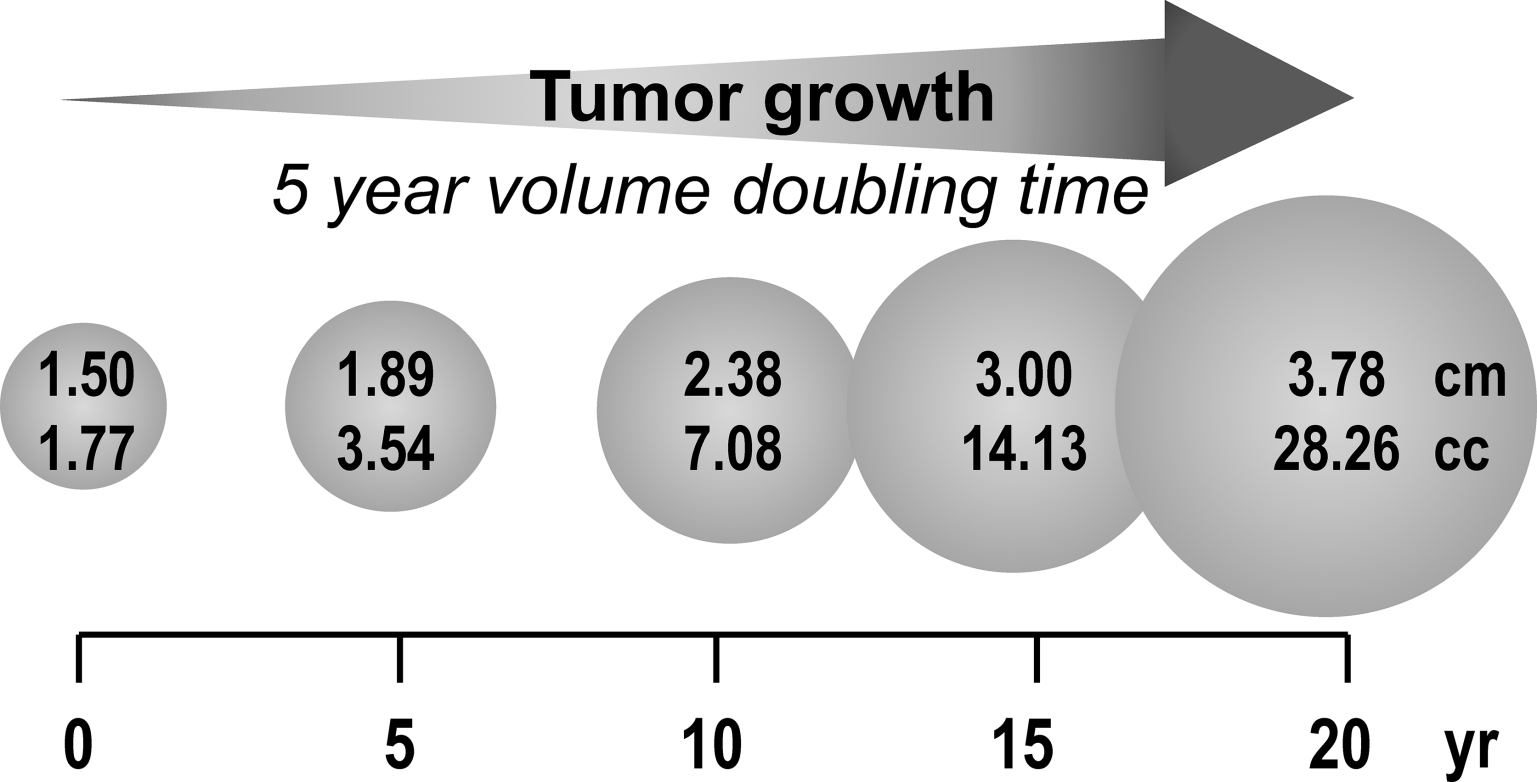
Estimated duration (years) for a 1.5 cm PPGL to reach 3 to 4 cm based on reported minimum tumor volume doubling time of 5 years.

It is also important to appreciate that PPGL are typically slow growing with a volume doubling time of 5-7 years (10, 11). From this it can be expected that it would take at least 15 years for a tumor to enlarge from 1.5 to 3 cm in diameter (**Figure 1**). Furthermore, in the early stages when tumors are less than 1.5 cm, they are unlikely to produce enough catecholamines for detection by standard biochemical tests let alone to evoke the signs and symptoms that may alert clinicians to the possibility of the tumor. Even when tumors do attain a size and level of catecholamine secretion sufficient to evoke signs and symptoms, there is usually considerable delay in recognizing the significance of this (12). Given that the median diameter of PPGL detected on the basis of signs and symptoms is 4 cm (13), it can be appreciated that for any PPGL first diagnosed in adults under an age of 30 years, the tumor is likely to have originated in childhood. The prevalence of childhood PPGL commonly cited in the literature is 10-20% (14–16); however, with the considerations outlined above, this likely represents an underestimate, particularly for patients with cluster 1 type gene mutations who have a median age of tumor diagnosis of 29 to 32 years (17).

As in adulthood the most important factor for an early diagnosis of PPGL in children and adolescents is attentiveness to the clinical clues of these tumors by pediatric caregivers. For familial cases involving offspring or siblings identified with germline mutations of tumor susceptibility genes an earlier diagnosis can be facilitated by enrolment into routine surveillance programs beginning as early as 5 years of age depending on the mutated gene (18–20).

## Modes of Clinical Suspicion

The initial mode of clinical suspicion of a PPGL is usually based on signs and symptoms of catecholamine excess. Signs and symptoms in children as in adults include hypertension, particularly paroxysmal hypertension, as well as palpitations, excessive sweatiness, headache, visual disturbances, pallor, anxiety, tremor, panic/anxiety, constipation, and nausea and/or vomiting (14, 21, 22). Among these signs and symptoms, any of a few may be present and mostly in episodes. Weight loss associated with a lowered body mass index and high heart rate are also useful to consider, in fact, more so than hypertension, which is relatively common in adults and of limited discriminatory value (23). High blood pressure in children and adolescents is, however, uncommon and in combination with any other signs and symptoms should always be considered as a potential indicator of a catecholamine-producing tumor. Nighttime sweatiness, polyuria and disturbances of vision or mental status have been reported in pediatric cases of PPGL (15), and might warrant particular attention.

As in adults, some children with PPGLs may be normotensive and asymptomatic (24), particularly when tumors are found as part of surveillance programs involving family members with a known mutation of a tumor susceptibility gene (25). Children with incidentally discovered adrenal or extra-adrenal masses based on imaging studies for reasons other than a suspected PPGL can also be normotensive and asymptomatic and have a catecholamine-producing tumor (26, 27).

For neuroblastoma, which have a more limited hereditary background compared to PPGL and do not usually secrete catecholamines in amounts sufficient to cause signs and symptoms, the mode of initial clinical suspicion is different from that for chromaffin cell tumors. Since routine screening for the tumors is now out of favor, neuroblastoma are usually suspected based on findings of a palpable abdominal mass or as a mass found incidentally during ultrasonography (28), including on occasion during prenatal ultrasound (29). Masses in the neck or thoracic regions may be discovered on the basis of Horner’s syndrome. Children with neuroblastoma may also present with fever, weight loss, bone or joint pain, or other symptoms that may evoke discovery during imaging studies. Hematological abnormalities from bone marrow involvement can also lead to discovery of these tumors and eventual diagnosis achieved by histopathology often after percutaneous needle biopsy.

## Catecholamine Synthesis, Metabolism and Secretion

Both PPGL and neuroblastoma are characterized by synthesis and metabolism of catecholamines within tumor cells. For any appreciation of the use of catecholamine-related biomarkers for diagnosis of these tumors it is useful to understand the pathways of catecholamine biosynthesis, storage, metabolism and secretion (30).

**Figure 2:**
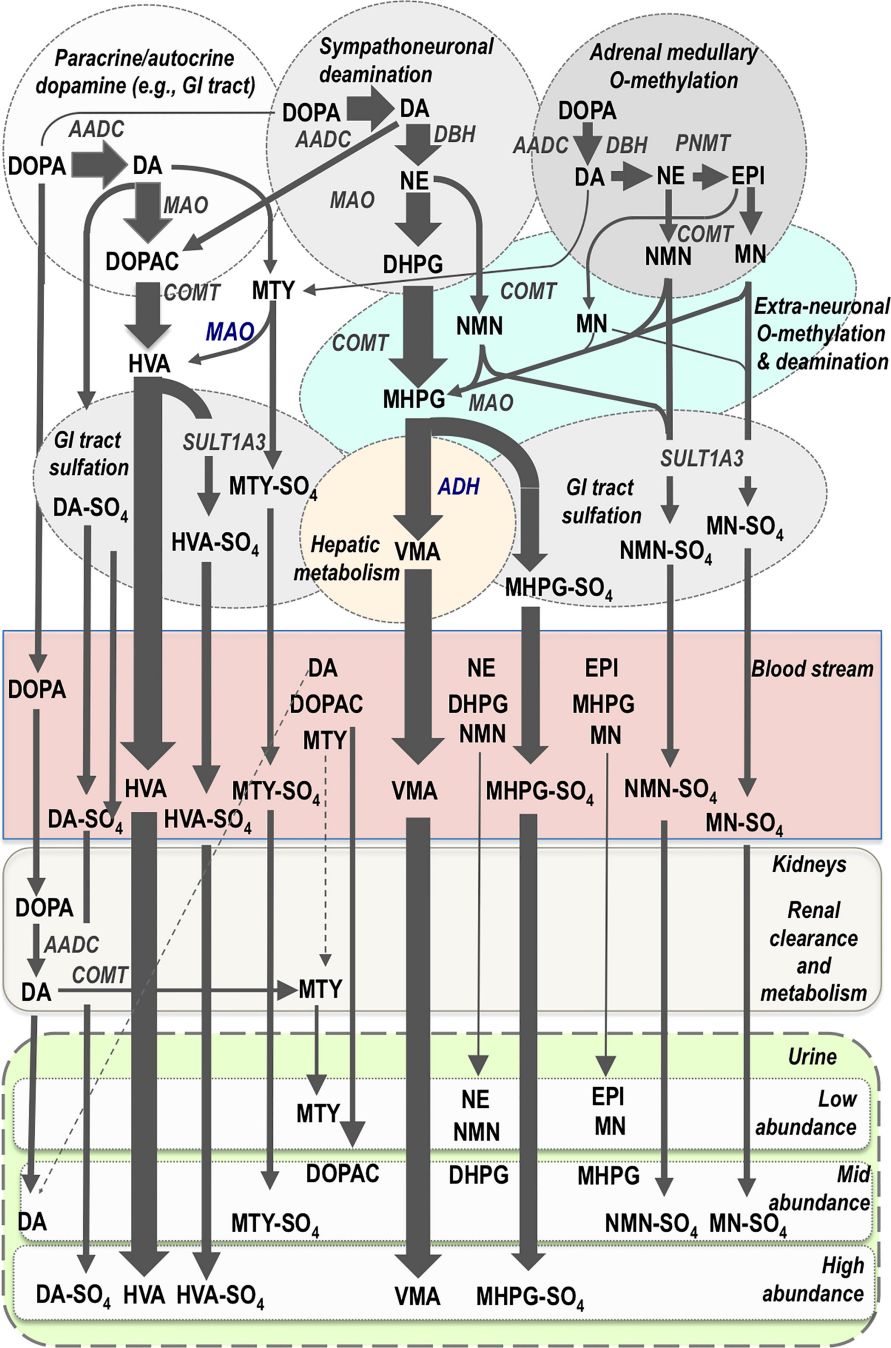
Pathways of catecholamine metabolism according to initial biosynthesis of dopamine (DA) in paracrine and autocrine systems, of DA and norepinephrine (NE) in sympathoneuronal systems and of DA, NE and epinephrine (EPI) in chromaffin cells of the adrenal medulla. Thereafter the main metabolic pathways are shown according different compartments of metabolism and according to entry into the bloodstream and thereafter clearance and metabolism by the kidneys for elimination in the urine. Flux through metabolic pathways is reflected by arrow size and final abundance of metabolites in the blood stream and urine. The normetanephrine (NMN), metanephrine (MN) and methoxytyramine (MTY) in the bloodstream are produced in low abundance, primarily from adrenal chromaffin cells, and thereby provide better diagnostic signals for PPGLs than metabolites produced in higher abundance and largely from sources other than adrenal chromaffin cells. Almost all catecholamines and their metabolites undergo some degree of sulfate conjugation in humans, but only the main sulfate-conjugated metabolites are displayed. Note also the high abundance of dopamine metabolites, despite their minor source from sympatho-adrenal medullary systems. AADC, aromatic amino acid decarboxylase; DBH, dopamine-beta-hydroxylase; PNMT, phenylethanolamine-N-methyltransferase; MAO, monoamine oxidase; COMT, catechol-O-methyltransferase; SULT1A3; sulfotransferase isoenzyme 1A3; ADH, alcohol dehydrogenase; DOPA, 3,4-dihydroxyphenylalanine; DOPAC, 3,4-dihydroxyphenylacetic acid; DHPG, 3,4-dihydroxyphenylglycol; MHPG, 3-methoxy-4-hydroxyphenylglycol; HVA, homovanillic acid; VMA, vanillylmandelic acid; MTY-SO_4_, methoxytyramine-sulfate; NMN-SO_4_, normetanephrine-sulfate; MN-SO_4_, metanephrine-sulfate; HVA-SO_4_, homovanillic acid-sulfate; MHPG-SO_4_, 3-methoxy-4-hydroxyphenylglycol-sulfate; DA-SO_4_, dopamine-sulfate.

Catecholamine biosynthesis starts with conversion of tyrosine to 3,4-dihydroxyphenylalanine (DOPA) by the rate limiting enzyme, tyrosine hydroxylase. DOPA is then converted to dopamine by aromatic-L-amino acid decarboxylase, an enzyme with a wide tissue distribution and broad substrate specificity. Dopamine is then transported by vesicular monoamine transporters into vesicular storage granules, where it is further converted to norepinephrine by dopamine β-hydroxylase, an enzyme with a unique presence in vesicular storage granules. Presence of phenylethanolamine N- methyltransferase (PNMT) in adrenal chromaffin cells leads to further conversion of norepinephrine to epinephrine (**Figure 2**); however, since PNMT is a cytosolic enzyme, this step depends on leakage of norepinephrine from vesicular storage granules into the cell cytoplasm. Epinephrine is then translocated back into storage granules from where it can be actively secreted as a circulating hormone.

Importantly and contrary to usual textbook depictions, vesicular stores of catecholamines do not exist in a static state until exocytotic secretion (31). Rather, vesicular stores of catecholamines exist in a highly dynamic equilibrium with the surrounding cytoplasm, with passive outward leakage into the cytoplasm counterbalanced by inward active transport under the control of vesicular monoamine transporters. The processes of active exocytotic secretion versus passive leakage of catecholamines from vesicular stores are entirely different and contribute differently to the metabolism of catecholamines produced at different sites including within tumor cells (32). These differences, however, are rarely appreciated despite the importance of this understanding for clinical applications of catecholamine-related biomarkers for diagnosis of catecholamine-producing tumors (30).

Exocytotic secretion of catecholamines involves the emptying of contents of vesicular catecholamine stores into the surrounding extracellular space. The process for hormonal secretion from the adrenal medulla involves closer proximity to the bloodstream than for norepinephrine secreted by sympathetic neurons, which acts locally rather than systemically. Close to 90% of norepinephrine secreted by sympathetic nerves is removed back into nerves by neuronal uptake so that only a small proportion escapes into the bloodstream or is metabolized at extra-neuronal locations before entry into the bloodstream (31). Some of the norepinephrine recaptured by sympathetic nerves is deaminated intraneuronally to 3,4-dihydroxyphenylglycol (DHPG) by monoamine oxidase (MAO), but most is returned to vesicular stores *via* vesicular monoamine transporters. By far the most DHPG is produced not after neuronal reuptake but rather after vesicular leakage of norepinephrine into the cytoplasm. DHPG is thereby the main initial metabolite produced from norepinephrine (**Figure 2**). DHPG is further metabolized at extraneuronal sites by catechol-O-methyltransferase (COMT) to 3-methoxy-4-hydroxyphenylglycol (MHPG). Thereafter most MHPG is metabolized in the liver by alcohol dehydrogenase to vanillylmandelic acid (VMA), the main urinary metabolic end-product of norepinephrine metabolism (33).

The processes of metabolism for catecholamines synthesized and secreted by adrenal chromaffin cells are somewhat similar to those for catecholamine-producing tumors, but entirely different from those for the norepinephrine produced in sympathetic nerves (31). First and foremost, while sympathetic nerves express only MAO, chromaffin cells also express COMT and thereby produce O-methylated metabolites (31). These include metanephrine from epinephrine, normetanephrine from norepinephrine and methoxytyramine from dopamine (**Figure 2**). The same former two metabolites can also be produced at extraneuronal locations from the catecholamines secreted into the bloodstream from the adrenals or from the norepinephrine secreted by sympathetic nerves. For circulating metanephrine, over 90% is derived from epinephrine metabolized within adrenal chromaffin cells rather than from epinephrine secreted from the same chromaffin cells (34). For circulating normetanephrine, about 25% is produced within adrenal chromaffin cells and 75% from extraneuronal metabolism of norepinephrine secreted by sympathetic nerves.

The processes for metabolism of dopamine are somewhat different in that substantial amounts of this catecholamine are not produced in the sympatho-adrenal system, but rather in diffuse paracrine systems of the gastrointestinal tract, kidneys and other tissues (35, 36) (**Figure 2**). In the kidneys, dopamine is produced after renal uptake and local metabolism of circulating DOPA (37). Thus, more than 90% of urinary dopamine is formed from circulating DOPA rather than circulating dopamine (38). Urinary methoxytyramine appears to be derived by similar processes that may also include renal O-methylation of the dopamine produced from circulating DOPA (39). Finally, the end-product of dopamine metabolism, homovanillic acid (HVA), is derived from the combined actions of COMT and MAO; unlike VMA, the production of HVA does not require any additional actions of hepatic alcohol dehydrogenase so that HVA has distinctly different sources from VMA (35).

Apart from final metabolism of catecholamines and catecholamine metabolites to HVA and VMA, all compounds also undergo varying degrees of sulfate conjugation (**Figure 2**). This process occurs mainly in gastrointestinal tissues, the site of expression of the required sulfotransferase isoenzyme, SULT1A3 (40). The sulfated metabolic end-products are primarily removed by the kidneys and excreted in urine. This is particularly important for dopamine and its metabolites, but also provides an important metabolic pathway for O-methylated catecholamine metabolites. Thus, the normetanephrine, metanephrine and methoxytyramine commonly measured in urine after acid hydrolysis mainly reflect sulfate conjugates that have partly different sources from the more rapidly cleared and thus much lower concentrations of circulating free metabolites (41).

## Tumoral Catecholamine Metabolism

**Figure 3:**
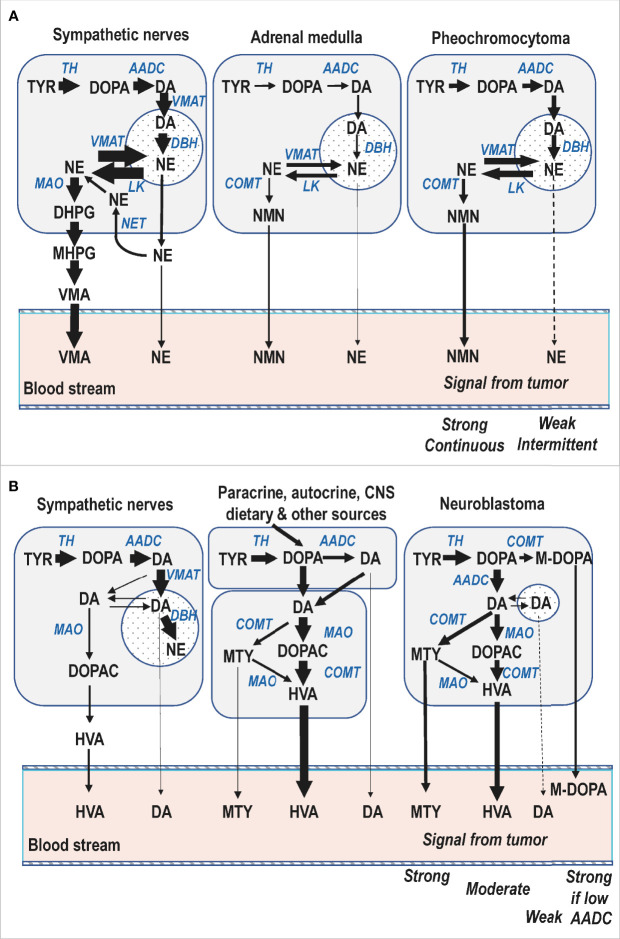
Simplified diagrammatic representations of catecholamine biosynthetic and metabolic pathways in a pediatric patient with a cluster 1 norepinephrine-producing PPGL **(A)** and another with a dopamine-producing neuroblastoma **(B)**. For both panels the storage vesicles where dopamine is converted to norepinephrine and from where catecholamines are secreted by exocytosis are depicted by the circular compartments. For neuroblastoma the smaller size of that compartment serves to illustrate lower numbers of vesicular storage granules and associated minimal catecholamine secretory activity of these tumors. AADC, aromatic amino acid decarboxylase; DBH, dopamine-beta-hydroxylase; MAO, monoamine oxidase; COMT, catechol-O-methyltransferase; VMAT, vesicular monoamine transporter; LK, leakage of catecholamines from vesicles; NET, cell membrane norepinephrine transporter; DOPA, 3,4-dihydroxyphenylalanine; M-DOPA, 3-O-methyl-dopa; DA, dopamine; NE, norepinephrine; DOPAC, 3,4-dihydroxyphenylacetic acid; DHPG, 3,4-dihydroxyphenylglycol; MHPG, 3-methoxy-4-hydroxyphenylglycol; HVA, homovanillic acid; VMA, vanillylmandelic acid; MTY, methoxytyramine; NMN, normetanephrine.

With the considerations outlined above concerning the subcellular, cellular and organ wide compartmentalized disposition of catecholamines it can be better appreciated why the O-methylated metabolites of catecholamines offer the best biomarkers of catecholamine-producing tumors, as also displayed according to the simplifications of **Figure 3**. For cluster 1 norepinephrine-producing noradrenergic PPGL typical of childhood (**Figure 3**, panel A), the tumor-derived signal for free normetanephrine commonly shows a stronger and larger proportional increase above normal plasma concentrations than for circulating norepinephrine, 90% of which is derived from sympathetic nerves (34). That large proportion serves to dilute the diagnostic signal from tumors considerably more than for circulating normetanephrine. Moreover, the tumoral production of normetanephrine is continuous, whereas the exocytotic secretion of norepinephrine by tumors can be intermittent or minimal unless provoked. The signal produced by VMA is also largely diluted by the considerable amounts of this metabolite originally derived from the DHPG produced in sympathetic nerves, which lack COMT. Similarly, the normetanephrine sulfate produced in gastrointestinal tissues from locally secreted normetanephrine dilutes the signal for this metabolite, thereby explaining lower diagnostic signal for urinary deconjugated normetanephrine than for free normetanephrine (42).

Similar considerations to those outlined above also clarify why measurements of plasma free normetanephrine provide a stronger diagnostic signal than urinary VMA for children with neuroblastoma (4). However, unlike PPGL, in neuroblastoma it is the dopamine metabolites and not the norepinephrine metabolites that provide the more consistently increased biomarkers of excess catecholamine production. The considerations for catecholamine metabolism in neuroblastoma are also somewhat different than in PPGL where COMT is expressed in larger abundance than MAO, the expression of which is also lower than in adrenal medullary chromaffin cells (43, 44). Neuroblastomas express not only COMT (45), but also MAO in high abundance (46), and thus produce significant amounts of both deaminated and O-methylated catecholamine metabolites (**Figure 3**, panel B). Nevertheless, since HVA is normally produced in substantial amounts by an array of different pathways, this production acts to dilute the diagnostic signal for this metabolite when produced by neuroblastoma. In contrast, free methoxytyramine circulates at very low plasma concentrations so the diagnostic signal for this metabolite is stronger than for urinary HVA (4). For some tumors plasma concentrations of DOPA and 3-O-methyldopa provide the only signal (4, 47), presumably due to their poorly differentiated nature, including minimal expression of aromatic-L-amino acid decarboxylase, the enzyme that converts DOPA to dopamine.

## Biochemical Tests of Catecholamine Excess

For neuroblastoma the value of measuring catecholamine metabolites as biomarkers, rather than the catecholamines themselves, was established in the 1970s from the observations of LaBrosse and colleagues (48). As shown by these investigators, neuroblastomas display a relative lack of the catecholamine storage vesicles characteristic of mature chromaffin cells and their PPGL derivatives. Thus, these tumors usually do not present with hypertension or increased plasma or urinary catecholamines, so that biochemical tests have traditionally depended on measurements of catecholamine metabolites, in particular HVA and VMA.

Intra-tumoral metabolism of catecholamines in PPGLs was also described in the 1960s by Crout and Sjoerdsma (49). Nevertheless, because PPGLs are characterized by hypertension and symptoms of catecholamine excess, those early findings were largely ignored and diagnosis continued to focus on measurements of catecholamines. It was not until after the turn of the 21^st^ century that emphasis moved from catecholamines to their O-methylated metabolites, the metanephrines (50). Shift in emphasis from catecholamines to metanephrines for diagnosis of PPGLs followed the understanding outlined earlier about how catecholamines are synthesized, stored and metabolized independently of secretion and by different pathways. Consequently, recommendations today mandate measurements of plasma free or urinary fractionated metanephrines as initial screening tests for PPGLs (51). Among these tests, plasma measurements offer superior diagnostic accuracy than urinary measurements (42). For the latter it should be appreciated that these may involve either the free metabolites or the more commonly used and much higher concentrations of sulfate-conjugated metabolites measured together with the free metabolites after an acid hydrolysis deconjugation step. The measurements of urinary free metabolites offer some diagnostic advantages over the combined free and deconjugated metabolites, though the value of urinary free methoxytyramine for identification of dopamine producing tumors is limited (42). Most likely this reflects origins of urinary free methoxytyramine from renal uptake, clearance and metabolism of circulating DOPA (**Figure 2**).

The vast majority of studies that have examined the diagnostic performance of biochemical tests for patients with PPGL have been in adults, with only a few isolated reports in children some of which employed outdated measurements of urinary VMA or spectrophotometric measurements of total metanephrines rather than fractionated normetanephrine and metanephrine (**Table 1**). Nevertheless, there have been two reports that documented high diagnostic accuracy of plasma free metanephrines for childhood cases of PPGL (25, 52).

**Table 1:** Diagnostic performance of biochemical biomarkers for pediatric PPGL.

Study reference	Sample matrix	Biomarker	Diagnostic sensitivity	Diagnostic specificity
Sarathi et al., *Endocr Prac* 2012;18:694-9 (52)	Plasma	NMN+MN	100% (17/17)	85.7% (67/78)
Weise et al.*, J Clin Endocrinol Metab* 2002;87:1955-60 (25)	Plasma	NMN+MN	100% (12/12)	94% (31/33)
		NE+EPI	92% (11/12)	91% (30/33)
	Urine	NMN+MN	100% (5/5)	95% (21/22)
		NE+EPI	100% (10/10)	83% (25/30)
Barontini et al., Ann NY Acad Sci 2006;1073:30-7 (98)	Urine	NMN	95% (55/58)*	No data
		MN	68% (39/58)*	No data
		NE	98% (57/58)*	No data
		EPI	45% (26/58)*	No data
		VMA	93% (54/58)*	No data
Perel et al., *Pediatr Hemat Oncol* 1997;14:413-22 (99)	Urine	MN	91.7% (11/12)*	No data
		VMA	95.7% (22/23)*	No data

*No information provided on reference intervals.

The two catecholamine metabolites that continue to provide the mainstay for biochemical testing of neuroblastoma are HVA and VMA, usually measured in urine. Since the HVA and VMA derived from neuroblastoma or PPGL are diluted by considerable amounts of the same metabolites produced from other sources, these metabolites are relatively poor diagnostic markers for catecholamine-producing tumors. For adult pheochromocytoma, diagnostic sensitivity of VMA reaches only to 46-77% compared to 97-99% for plasma free metanephrines at similar specificities (53). In a prospective trial involving 1.5 million neonates only 73% of all infants detected at follow-up with neuroblastoma had elevated urinary excretion of HVA or VMA at screening (54). Moreover, many that were detected by screening were those that spontaneously regressed while those that were missed were usually aggressive. Consequently, screening programs involving urinary HVA and VMA have been abandoned. Today, diagnosis of neuroblastoma depends primarily on biopsy-dependent histopathology, which combined with genomic biomarkers (e.g., *MYCN* amplification) can provide information for staging and therapeutic intervention (55).

Reflecting historical precedence, almost all reports that have examined the utility of tests of catecholamine excess to identify children with neuroblastoma have included measurements of urinary HVA and VMA (**Table 2**). Nevertheless, there have been recent reports that have examined utility of plasma free or urinary measurements of normetanephrine and methoxytyramine. Thus, similar to PPGL, there is now evidence that measurements of plasma normetanephrine and methoxytyramine provide excellent biomarkers for identification of patients with neuroblastoma (4, 56, 57), including one study that showed the expected superiority over urinary HVA and VMA (4). Introduction of new biochemical tests for neuroblastoma is, nevertheless, largely made futile by reliance on biopsy-dependent histopathological diagnosis and measurements of urinary HVA and VMA for assessing tumoral catecholamine production.

**Table 2:** Diagnostic performance of biochemical biomarkers for neuroblastoma.

Study Reference	Sample matrix	Biomarker	Diagnostic sensitivity	Diagnostic specificity
Peitzsch et al., *Pediatr Blood Cancer* 2019;e28081 (60)	Plasma	NMN	72.9% (70/96)^1^	97.6% (40/41)^1^
		MTY	96.8% (93/96)^1^	97.6% (40/41)^1^
		NMN+MTY	97.9% (94/96)^1^	95.1% (39/41)^1^
	Urine	VMA	73.2% (60/82)	84.8% (28/33)
		HVA	75.6% (68/90)	87.9% (29/33)
		VMA+HVA	82.2% (74/90)	84.8% (28/33)
Barco et al., *Clin Biochem* 2019;66:57-62 (57)	Plasma	NMN	80.4% (43/54)^2^	100%^2^
		MTY	88.2% (48/54)^2^	95.8%^2^
		NMN+MTY	92% (50/54)^2^	92%^2^
	Urine	VMA	79% (30/38)^2^	No data
		HVA	90% (34/38)^2^	No data
Franscini et al., *Pediatr Blood Cancer* 2015;62:587-K2793 (56)	Plasma	NMN	100% (10/10)	No data
		MTY	100% (10/10)	No data
Hwang et al., *Molecules* 2021;26:3470 (100)	Urine	VMA	75% (15/20)^3^	No data
		HVA	85% (17/20)^3^	No data
		VMA+HVA	90% (18/20)^3^	No data
Verly et al., *Eur J Cancer* 2017;72:235-43	Urine	NMN	89% (268/301)^4^	No data
		MTY	69% (208/301)^4^	No data
		NMN+MTY	92% (277/301)^4^	No data
		VMA	66% (199/301)^4^	No data
		HVA	82% (247/301)^4^	No data
		VMA+HVA	84% (253/301)^4^	No data
Barco et al., *Clin Biochem* 2014;47:848-52 (57)	Urine	VMA	81.6% (146/179)^2^	93.8%^2^
		HVA	80.5% (136/169)^2^	92.3%^2^
		VMA+HVA	81.9% (130/159)^2^	91.2%^2^
Strenger et al., *Pediatr Blood Cancer* 2007;48:504-9 (101)	Urine	VMA	80.7% (92/114)^5^	No data
		HVA	71.9% (82/114)^5^	No data
		DA	61.3% (70/114)^5^	No data
		VMA+HVA	88.6% (101/114)^5^	No data
		VMA+HVA+DA	91.2 (104/114)^5^	No data
Monsaingeon et al., *Eur J Pediatr* 2003;162:397-402 (102)	Urine	NMN	96.6% (28/29)^2^	97%^2^
		VMA	96.6% (28/29)^2^	96%^2^
		HVA	93.1% (27/29)^2^	99%^2^
Schilling et al., *NEJM* 2002;346:1047-53 (54)	Urine	VMA +HVA	73% (109/149)	99.8% (1,470,864/1,472,469)
Candito et al.*, Med Pediatr Oncol* 1992;20:215-20 (103)	Urine	NMN	72% (13/18)	No data
		MTY	89% (16/18)	No data
		VMA	78% (14/18)	No data
		HVA	67% (12/18)	No data
Tuchman et al., *Pediatrics* 1987;79:203-5 (104)	Urine	VMA	72.5% (29/40)^6^	No data
		HVA	90% (36/40)^6^	No data
LaBrosse et al., *Cancer Res* 1980;40:1995-2001 (105)	Urine	VMA	71% (151/213)^7^	No data
		HVA	75% (159/213)^7^	No data

1) Reference intervals from Peitzsch et al., Clin Chim Acta 2019;494:100 (60).

2) performance characteristics derived from ROC analysis.

3) Reference intervals from Rifai, N. Tietz Textbook of Clin Chem Mol Diag, 6th ed.; Elsevier: Louis, MO, USA, 2018 (106).

4) Reference intervals from Davidson et al., Ann Clin Biochem 2011;48(Pt 4):358 (63).

5) Reference intervals from Kerbl et al., Eur J Cancer 1996;32A:2298 (107).

6) Reference intervals from Tuchman et al., Pediatrics 1985;75:324 (108).

7) Reference intervals from Gitlow et al., J Lab. Clin Med 1978;72:612 (109), Voorhess, Pediatrics 1967;39:252 (110).

Urinary rather than plasma methoxytyramine has also recently been advanced as an alternative to urinary HVA for identification of neuroblastoma (58). This metabolite, similar to urinary dopamine, appears to be largely derived from DOPA and is thus not a particularly good biomarker of tumoral dopamine metabolism (42). Nevertheless, these measurements appear to have prognostic utility (5). Since plasma DOPA also shows prognostic utility in neuroblastoma (47, 59), it is possible that this may underly the prognostic utility of urinary methoxytyramine. With prognostic rather than diagnostic utility in mind, there may be a rationale to advance from current antiquated reliance on urinary HVA and VMA to new and improved methods for biochemical testing of neuroblastoma.

## Reference Intervals

The need to establish reference intervals for biochemical tests of catecholamine excess represents another impediment to moving from outdated methods of diagnosis to new and improved biochemical tests to detect catecholamine-producing tumors. For adults it is relatively simple to obtain blood or urine specimens to establish reference intervals and further test utility of those reference intervals in patient populations. The practical and ethical barriers to procure blood or urine specimens for establishing reference intervals are more complex to negotiate for pediatric than adult populations (60). Consequently, advances in place for adults can be considerably delayed to implement for children.

**Figure 4:**
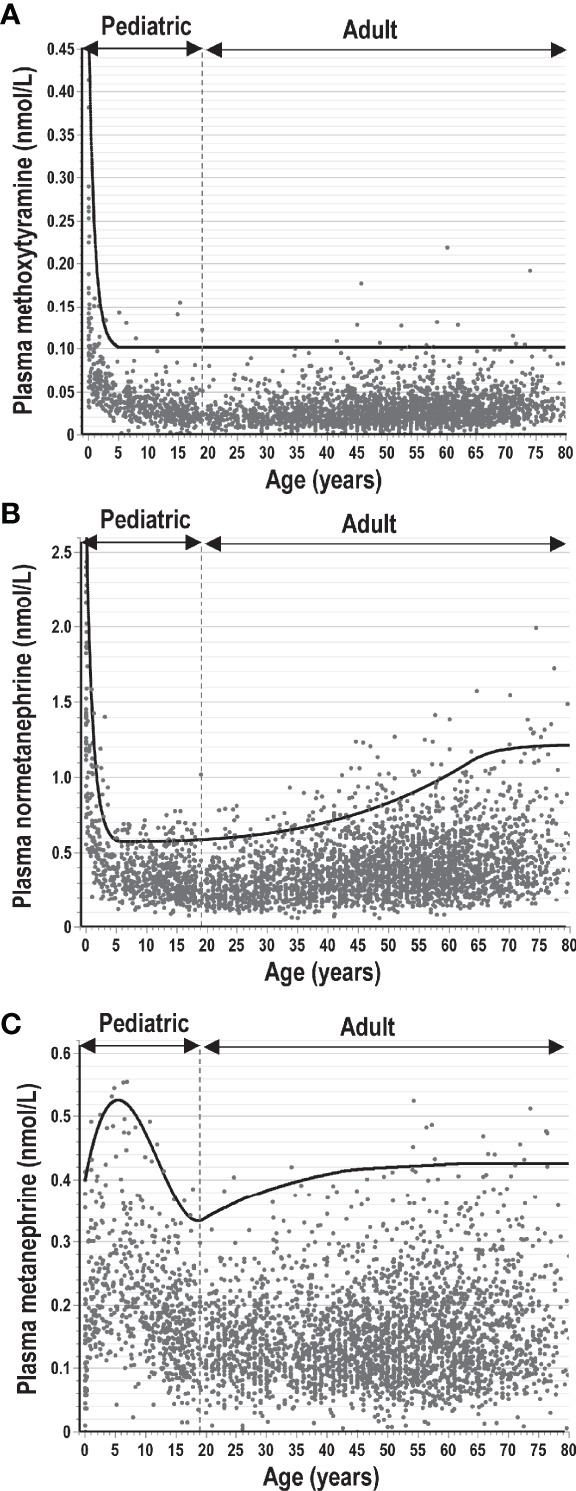
Plasma concentrations of methoxytyramine **(A)**, normetanephrine **(B)** and metanephrine **(C)** from infancy and early childhood through adolescence to later ages in adults. Concentrations are derived from measurements in 3706 individuals (including 707 children from 0-18 years of age) without catecholamine producing tumors according to previously published data (4, 25, 42, 60). The solid lines indicate upper limits of reference intervals, which for adults and children over 5 years of age were optimized to provide optimum diagnostic sensitivity for normetanephrine at optimal specificity and lower proportions of false-positive results (<1%) for metanephrine and methoxytyramine.

As with many biomarkers, those involving tests of catecholamine excess can show variable differences between adults and children and within the pediatric population differences according to developmental age and sex. For plasma free normetanephrine and methoxytyramine there are highly dynamic changes in early childhood with markedly higher plasma concentrations in neonates that drop rapidly within the first year (**Figure 4**). Thereafter, concentrations level off, though for normetanephrine slowly climb later in adolescence and continue to increase throughout adulthood. In contrast, plasma concentrations of metanephrine increase during early infancy, are higher in young children than adults and remain higher in males than females (25). The reciprocal changes of methoxytyramine and normetanephrine compared to metanephrine in early childhood are suggested to reflect apoptosis of neural crest-derived cells of the paraganglia from which the former metabolites derive, changes that contrast with development of the adrenal chromaffin cells responsible for almost all circulating metanephrine (60). For use of O-methylated catecholamine metabolites as biomarkers of neuroblastoma it is essential to employ age-specific reference intervals, which can be achieved from polynomial curve fitting (56, 60). For children over 5 years of age, plasma concentrations of methoxytyramine and normetanephrine remain relatively constant throughout childhood and all that is required are reference intervals for that broader age range. For metanephrine, concentrations are higher in boys than girls and particularly in younger children, when slightly higher cut-offs may be preferable compared to adolescents and adults.

**Figure 5:**
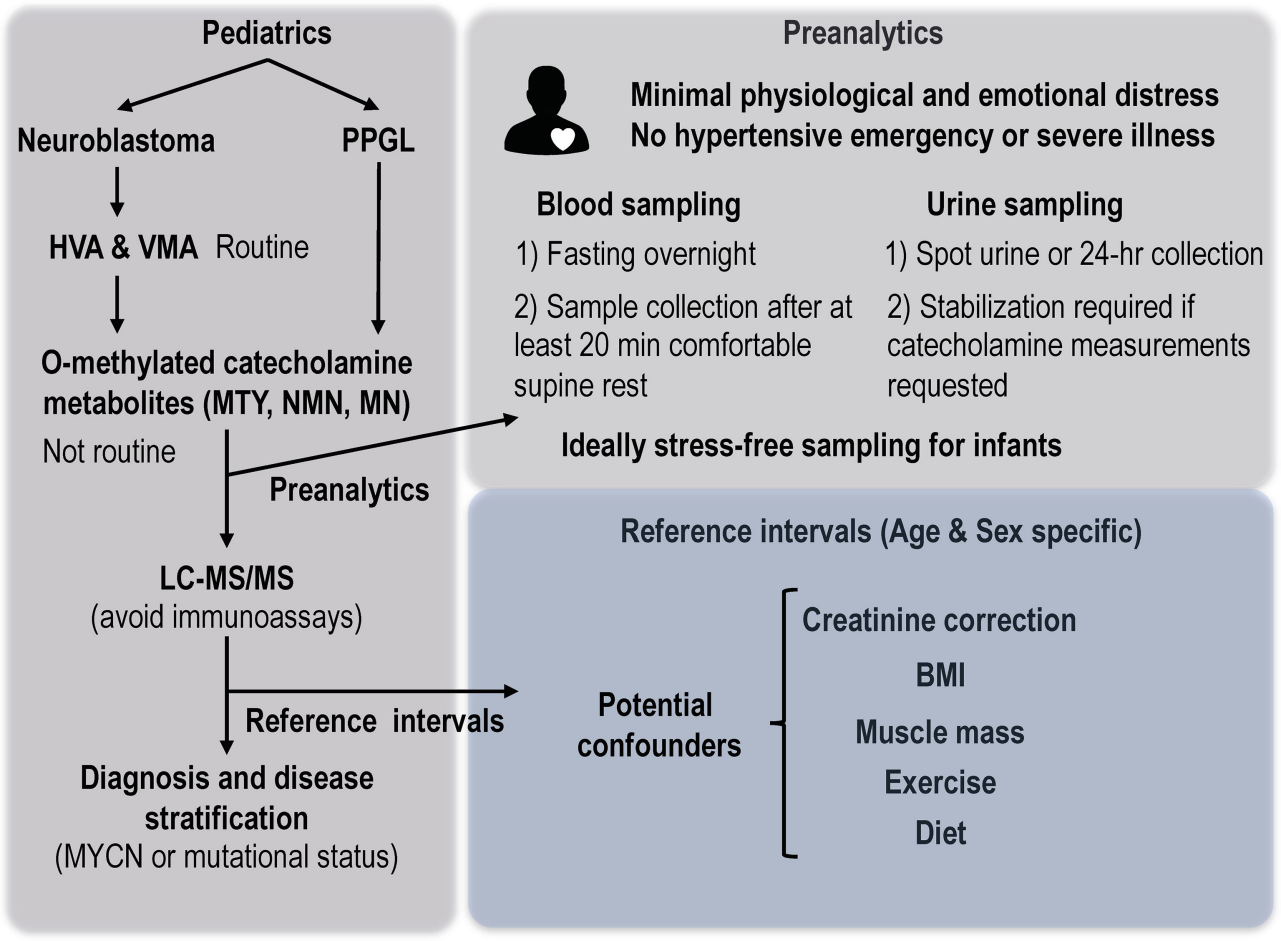
Considerations of preanalytics and reference intervals for interpretation of biochemical tests of catecholamine excess in children. MTY, methoxytyramine; NMN, normetanephrine; MN, metanephrine; BMI, body mass index.

Since diagnosis of catecholamine-producing tumors of childhood have historically involved measurements in urine, reference intervals for this sample matrix are those that have received the most attention (61–65). However, such measurements are not without associated problems for establishing reference intervals. Since 24-hr collections of urine from both infants and older children are unreliable and difficult, spot urines are the method commonly employed with dilutional differences corrected using creatinine (61). This, however, has the problem of introducing another variable as a denominator (**Figure 5**). Urinary outputs of creatinine vary according to diet, exercise and most importantly muscle mass (66, 67). Consequently, urinary outputs of creatinine are higher in males than females and are positively correlated to body mass or body surface area and increase substantially from early infancy throughout childhood (67–69). There are also body size related increases in urinary outputs of catecholamines and their metabolites, but these are not as substantial as those for creatinine. Thus, although 24-hr urinary outputs of catecholamines and metabolites increase throughout childhood, when expressed as a ratio to urinary outputs of creatinine there are marked decreases throughout childhood and particularly in infancy (61–65).

Due to the dynamic changes in urinary excretion of both creatinine and catecholamine metabolites, reference intervals must be established for different age groups (61–65). Thus, for example, as reported by Davidson et al. (63), upper limits for urinary excretion of normetanephrine as a ratio to creatinine vary considerably from 0.529 nmol/mol for infants below one year to 0.123 nmol/mol for children 8 to 10 years of age and 0.086 nmol/mol for those between 14 to 19 years of age. As reported by Cole et al. (61), use of creatinine to correct for dilutional influences on measured concentrations of VMA and HVA in spot urine samples suffers from several limitations that impact reliability of measurements as a laboratory test for neuroblastoma. These investigators proposed further adjustments for sex and body weight.

For all reference intervals due consideration must also be given to the methods of measurement. Early spectrophotometric methods as well as more recent immunoassay methods can be inaccurate and suffer from interferences so that reference intervals developed by these methods can be unreliable (70). Even for techniques employing gas or liquid chromatographic separation there can be differences in measurements between laboratories so that reference intervals should be validated by each laboratory and not simply involve those reported in the literature. Mass spectrometric methods offer opportunities for both improved analytical specificity and accuracy. By participation in interlaboratory proficiency programs or comparison studies there is now the possibility for both harmonized measurements and reference intervals (71).

## Preanalytical Considerations

Apart from appropriately established reference intervals and accurate measurement methods, consideration of preanalytics and other patient specific variables is crucial to interpretation of analytical test results (72), particularly those that fall close to either side of upper limits of reference intervals. For both plasma and urinary measurements, it is important that samples are procured with patients under conditions of minimal physiological or emotional distress (**Figure 5**). In adults it is well established that emergency situations and acute illness can result in plasma concentrations or urinary outputs of metanephrines that can be indistinguishable from those in patients with catecholamine-producing tumors (73). The same can also be expected for children, so that ideally testing for a catecholamine-producing tumor should be undertaken after recovery from a hypertensive emergency or severe illness.

Exercise or other forms of physical activity should be avoided or minimized before blood collections or preceding and during urine collections. First morning urine collections are associated with lower urinary outputs of catecholamines and metabolites due to the preceding nighttime rest and have been proposed as a means to minimize false-positive results (74). However, as yet this has not been verified for diagnosis of catecholamine-producing tumors in childhood.

For measurements of plasma metanephrines it is important that blood samples are taken after at least 20 minutes of comfortable supine rest, but for young children this can be difficult and requires close supervision (25). Similarly, although it is standard practice to acquire blood samples by direct venipuncture, this is established in adults to result in higher plasma concentrations and rates of false-positive results than sampling using a previously inserted intravenous cannula (75). For children, the emotional distress of venipuncture can be expected to be more troublesome than for adults. With preanalytical precautions in place high diagnostic sensitivity is preserved and false-positive results are minimal according to upper limits of reference intervals appropriately determined for children (25).

For younger children, stress-free blood sampling can be highly problematic, which represents a limitation of plasma compared to the urinary measurements typically used when testing children for suspected neuroblastoma. The volume of blood required can also be a limiting factor for neonates. However, new advances in sample preparation procedures before mass spectrometry is now allowing accurate and precise measurements of plasma methoxytyramine and other O-methylated metabolites in as little as 50 µl of plasma (76). With such advances it might even be possible to obtain blood by heel prick, which might provide an advantage of a blood versus a urine test.

Dietary influences and medications can also be important to consider. Certain foods can increase plasma concentrations of methoxytyramine as well as urinary outputs of catecholamine metabolites (77). For blood sampling such influences can be avoided by an overnight fast; however, for infants and urine collections, dietary restrictions may be preferable or more practical (60). Drugs that significantly impact monoamine systems, such as norepinephrine uptake blockers, can be troublesome in adults but are rarely administered to children. Although some antihypertensives and other medications can result in analytical interferences for some methods of analysis, such drugs are not usually troublesome with modern mass spectrometric methods (78).

## Biochemical Test Interpretation

Apart from assessment of whether test results are positive or negative, the pattern and nature of increases of the three O-methylated catecholamine metabolites can provide other information about a catecholamine producing tumor that cannot be so easily gleaned from measurements of the catecholamines themselves or other metabolites such as HVA and VMA. Extents and patterns of increases above reference intervals not only allow assessments of relative probabilities of a tumor, but also other information such as location, size, disease aggressiveness and type of disease-causing mutations (79, 80). For neuroblastoma the information is a little more limited than for PPGL, but from increases in methoxytyramine can be used to assess likelihood of *MYCN* amplification (4, 5), which has prognostic significance.

For PPGL, increases in plasma methoxytyramine and metanephrine can be used to assess likelihood of extra- versus intra-adrenal tumor locations (80), the former more common in pediatric than adult patients. Increases in methoxytyramine relative to normetanephrine can also be used to assess risk of metastatic disease (79), which as in neuroblastoma may also relate to the developmental aspects of the tumors and associated degree of differentiation. Related to this, relative increases of the three metabolites can be used to assess probabilities of underlying mutations in groups of genes (80), these differing in prevalence among children and young adults compared to older adults with the tumors (7, 8).

Magnitudes of increases above normal of all combined O-methylated metabolites correlate with disease burden and can thereby be used to predict tumor size (80). The generally slow growing nature of PPGLs is also reflected by time-dependent increases in plasma concentrations of the O-methylated metabolites, which can be useful in follow-up to better confirm a PPGL. This can be particularly useful in surveillance programs, such as in children with identified mutations of the von-Hippel Lindau (*VHL*) gene whereby relative changes over time can signal development of a PPGL better than a single point measurement (25).

## Differential Diagnosis

Neuroblastoma is more common in children than PPGL and since clinical presentations can overlap, there can be difficulties in the differential diagnosis of the two tumor entities (22). This difficulty has been highlighted in this issue by a case series of five children with PPGL who were initially diagnosed mistakenly with neuroblastoma, including some who underwent biopsy and all of whom were inadequately prepared before surgical intervention (81). Though commonly employed for neuroblastoma, percutaneous biopsy of a PPGL is potentially dangerous and is therefore contraindicated for those tumors (82, 83). Furthermore, because all patients, including children, with PPGL should be prepared for surgery using adrenergic blocking drugs (22, 84), it is imperative that there is no mistaking a PPGL for a neuroblastoma before scheduling fine needle biopsy or surgical intervention. Adding to the difficulties in differential diagnosis, cases have also been reported of composite neuroblastoma and pheochromocytoma (85–87). Furthermore, some gene mutations, such as those impacting the *myc-associated factor X* (*MAX*) gene, can predispose to both childhood neuroblastoma and pheochromocytoma (88, 89).

For PPGL at least, identification of disease depends crucially on biochemical tests of catecholamine excess; thus, if there is any doubt about a presumed neuroblastoma, the same tests should be considered in those cases. In the case series presented in this issue, involving five patients with PPGL and a mistaken initial diagnosis of neuroblastoma, only two underwent any form of testing for catecholamine excess (81). The problem remains, however, whether such tests can be used to distinguish neuroblastoma from PPGL.

Since both PPGL and neuroblastoma show overlapping patterns of increases in plasma dopamine and norepinephrine metabolites, as well as in some cases the precursor catecholamines themselves, these measurements alone are unlikely to offer a solution for differential diagnosis in all pediatric cases of these tumors. Plasma concentrations of 3-O-methydopa are negligibly impacted in patients with PPGL (personal observations), but show increases above cut-offs in about 75% of patients with neuroblastoma (4). Such increases may therefore be useful for distinguishing neuroblastoma from PPGL, but cannot solve the problem for more fully differentiated cases of neuroblastoma. As outlined earlier, neuroblastoma differ from PPGL in their relative expression of the primary catecholamine-metabolizing enzymes, MAO and COMT, with the former deaminating enzyme more highly expressed in neuroblastoma than PPGL. Thus, it is possible that, in addition to measurements of 3-O-methyldopa, different patterns of deaminated and O-methylated metabolites might be useful for differential diagnosis. This, however, remains to be determined.

Technologies such as those involving circulating tumor cells or DNA and protein biomarkers offer potential molecular diagnostic strategies for therapeutic stratification and monitoring in neuroblastoma (90, 91). Such approaches might also be also useful for differential diagnosis. Nevertheless, it will take considerable time to develop and establish liquid biopsies for these purposes, let alone prospectively determine any clinical utility, particularly in pediatric solid tumors (92).

Differences in imaging features between neuroblastoma and PPGL, such as presence of calcifications in the former, could also offer clues to help distinguish the two types of tumors (93). However, calcifications can occasionally occur in PPGL (94). Imaging characteristics of encasement, invasion and infiltration are relatively common in neuroblastoma (95), but far less so in PPGL and might thereby offer other clues to differentiate the two tumor types. Nevertheless, as indicated in the case series covered in this issue, there can be exceptions. As yet any use of functional imaging for differential diagnosis appears undocumented. Therefore, without histopathological confirmation, differential diagnosis of neuroblastoma and PPGL must continue to rely on a combination of considerations, including presentation and clinical features of the patient, imaging characteristics as well as whatever laboratory tests may be available.

## Biochemical Testing in Disease Surveillance Programs

While surveillance programs for neuroblastoma have all but disappeared, those for PPGLs are becoming increasingly important due to recognition of the high hereditability of the tumors. For families with identified germline mutations in tumor susceptibility genes, screening is recommended to start in childhood as early as 5 years for some gene mutations, such as for *succinate dehydrogenase subunit B* (*SDHB*) and *VHL*, that lead to activation of pseudohypoxia pathways (18, 19). A slightly later start at about 10 years is currently recommended for mutations of other pseudohypoxia genes and later still for mutations of genes impacting kinase signaling pathways, such as those encoding the rearranged during transfection proto-oncogene (*RET*) and the neurofibromatosis type 1 (*NF1*) gene (96, 97).

Since mutations of genes such as *VHL* and *SDHB* that result in activation of pseudohypoxia pathways lead to PPGLs that do not produce epinephrine, the focus of biochemical testing in children as in adults with these mutations should be on normetanephrine. However, for mutations of succinate dehydrogenase subunit genes, the resulting tumors often also produce methoxytyramine and sometimes only methoxytyramine (80). Therefore, for these children biochemical testing should include measurements of methoxytyramine, which for assessing tumoral dopamine production should involve measurements in plasma rather than urine (42). On the other hand, for children with mutations of *RET*, *NF1* or other genes that primarily impact kinase signaling pathways, interpretation of biochemical tests must include measurements of metanephrine since the associated tumors express PNMT and invariably produce epinephrine (80).

For all surveillance involving children, reference intervals established for pediatric populations are essential. With plasma measurements, use of reference intervals established for adults is likely to lead to false-negative results when applied to children over 5 years of age. This is particularly important for normetanephrine, which shows higher plasma concentrations in adults than in children (25).

## Future Perspective

While biochemical tests of catecholamine excess in neuroblastoma are of limited importance for diagnosis compared to biopsy based histopathological diagnosis and molecular analyses, there are situations where biopsy is either not possible or may be contra-indicated. For such situations biochemical tests of catecholamine excess remain useful, but for the most part continue to rely on measurements of VMA and HVA, tests that have long been abandoned for PPGL. There are now better tests of catecholamine excess than urinary HVA and VMA, but it will likely take considerable time, if ever, before these will enter the clinical mainstream for neuroblastoma. For PPGL such tests have been widely available for the past two decades, but still need to be better integrated into pediatric care. The need for appropriate transition of pediatric to adult care for childhood cases of PPGL is another challenge, and particularly important in light of the high rate of disease recurrence and/or metastasis for tumors that originate in childhood. PPGL, however, remain rare tumors and patient management requires a depth of knowledge not within reach for most pediatricians who might first encounter affected children. Appropriate management of all patients with PPGL, but especially those in childhood, requires a multidisciplinary approach. This need is now recognized by patient-led organizations that are establishing procedures for review and accreditation of centers with appropriate resources and expertise. Through these and other efforts it is possible that earlier diagnosis of PPGL might be achieved and the true prevalence of childhood PPGL clarified. On the other hand, biochemical tests of catecholamine excess in children with neuroblastoma are likely to follow the trend of the past six decades and continue to remain sub-optimally based on historical precedence rather than more ideally on contemporary understanding.

## Author Contributions

GE drafted the first version of manuscript, while MP, NB and AH contributed to the editing and revision of the manuscript. All authors provided conceptual input and MP additionally assisted with compilation of data from the literature.

## Funding

The authors gratefully acknowledge support from the Deutsche Forschungsgemeinschaft (DGE, 314061271-TRR/CRC 205 to GE, MP, NB and AH).

## Conflict of Interest

The authors declare that the research was conducted in the absence of any commercial or financial relationships that could be construed as a potential conflict of interest.

## Publisher’s Note

All claims expressed in this article are solely those of the authors and do not necessarily represent those of their affiliated organizations, or those of the publisher, the editors and the reviewers. Any product that may be evaluated in this article, or claim that may be made by its manufacturer, is not guaranteed or endorsed by the publisher.

## Abbreviations

PPGL, pheochromocytoma and paraganglioma; LC-MS/MS, liquid chromatography with tandem mass spectrometry; VMA, vanillylmandelic acid; HVA, homovanillic acid; PNMT, phenylethanolamine N-methyltransferase; COMT, catechol-O-methyltransferase; MAO, monoamine oxidase; DHPG, 3,4-dihydroxyphenylglycol; MHPG, 3-methoxy-4-hydroxyphenylglycol; MAX, myc-associated factor X; VHL, von-Hippel Lindau; SDHB, succinate dehydrogenase subunit B; RET, rearranged during transfection proto-oncogene; NF1, neurofibromatosis type 1.
